# Current Applications of Machine Learning for Spinal Cord Tumors

**DOI:** 10.3390/life13020520

**Published:** 2023-02-14

**Authors:** Konstantinos Katsos, Sarah E. Johnson, Sufyan Ibrahim, Mohamad Bydon

**Affiliations:** 1 Department of Neurologic Surgery, Mayo Clinic, Rochester, MN 55902, USA; 2 Mayo Clinic Neuro-Informatics Laboratory, Department of Neurologic Surgery, Mayo Clinic, Rochester, MN 55902, USA

**Keywords:** machine learning, artificial intelligence, deep learning, spinal cord tumors

## Abstract

Spinal cord tumors constitute a diverse group of rare neoplasms associated with significant mortality and morbidity that pose unique clinical and surgical challenges. Diagnostic accuracy and outcome prediction are critical for informed decision making and can promote personalized medicine and facilitate optimal patient management. Machine learning has the ability to analyze and combine vast amounts of data, allowing the identification of patterns and the establishment of clinical associations, which can ultimately enhance patient care. Although artificial intelligence techniques have been explored in other areas of spine surgery, such as spinal deformity surgery, precise machine learning models for spinal tumors are lagging behind. Current applications of machine learning in spinal cord tumors include algorithms that improve diagnostic precision by predicting genetic, molecular, and histopathological profiles. Furthermore, artificial intelligence-based systems can assist surgeons with preoperative planning and surgical resection, potentially reducing the risk of recurrence and consequently improving clinical outcomes. Machine learning algorithms promote personalized medicine by enabling prognostication and risk stratification based on accurate predictions of treatment response, survival, and postoperative complications. Despite their promising potential, machine learning models require extensive validation processes and quality assessments to ensure safe and effective translation to clinical practice.

## 1. Introduction

Spinal cord tumors are a diverse group of rare neoplasms that arise from tissues in and around the spinal canal, which tend to have an indolent onset with a gradual progression of signs and symptoms [1,2]. Similar to their intracranial counterparts, spinal cord tumors are characterized by histological heterogeneity, reflecting potential origination from a multitude of precursor cells. By convention, spinal cord tumors are commonly classified by anatomic sublocation as intradural intramedullary, intradural extramedullary, or extradural [1]. Surgical resection constitutes the mainstay approach for their definitive diagnosis and removal. For invasive tumors, where complete resection and local control are not feasible, adjuvant therapies are commonly implemented. Given the significant clinical and operative challenges associated with spinal cord tumors, physicians could benefit from insights that could potentially aid decision making and improve patient outcomes. However, while substantial progress has already been made in applying artificial intelligence-based techniques in brain tumors, the growing trend of these soft computing approaches has only recently been implemented in diagnosing and managing spinal cord tumors. In this manuscript, we aim to report and evaluate the current and potential applications of machine learning in spinal cord tumors.

### 1.1. Machine Learning

Machine learning represents a subset of artificial intelligence in which machines are able to “learn” from data without any explicit programming [3]. The features of the input data determine the output generated by the machine learning model. Therefore, machine learning possesses the ability to efficiently process vast amounts of information, which could potentially aid in diagnosing and managing complex diseases. Common applications of machine learning include:

- Classification: the input is allocated to a specific category among a group of two or more. For instance, machine learning models in spinal pathology could be implemented to determine disc degeneration severity, according to Pfirrman grading, by automatically assigning a magnetic resonance imaging scan of the disc to a category ranging from 1 to 5. Similarly, classification can be employed for image segmentation by labeling pixels based on anatomical regions and regional characteristics [4];
- Regression: the output generated is continuous. For example, determining the coordinates of a region of interest in a magnetic resonance scan would require regression techniques [4];
- Clustering: enables inputs to be assigned to groups by factoring features learned from the inputs themselves, allowing classification of data in the absence of prior knowledge [4].

Machine learning techniques can also be described in terms of the nature of the tasks performed:

- Supervised learning utilizes a dataset with predictor variables for which the correct output is known and establishes associations between the two [5]. Supervised techniques allow the identification of the optimal connections between input and output data by accurately modeling the difference between the machine predictions and the correct output [4]. Supervised learning is heavily used in the medical field;
- Unsupervised learning uses input data for which the output is unknown. Such algorithms analyze unlabeled datasets to discover hidden patterns and extract new knowledge without the need for human intervention. Clustering constitutes a form of unsupervised learning.

Importantly, for any machine learning model to achieve the desired performance, it must appropriately pair the input data and their relative output during training. When the mapping function is determined, and if the training examples are sufficient, the model can accurately process new inputs similar to those included in the training. If the training dataset is insufficient, the model may generate results that fit the training data precisely but cannot make accurate predictions on new inputs (overfitting); or may not establish a complex enough function to capture the features of the input data (underfitting) [6].

Multiple methods are used for supervised learning and embody a cardinal component of the machine learning tasks employed in medical research. Linear regression, a commonly used method, formulates inputs as multidimensional vectors and maps them to the corresponding output. Its simplicity and inability to capture nonlinear behaviors make linear regression models more susceptible to underfitting and limit their applicability [4]. Logistic regression interprets inputs to a binary output according to the logistic sigmoid function, which represents the probability of the input corresponding to the “1” or “0” output [4]. Support vector machines are based on the notion that data points can be represented as points in a multidimensional space. Support vectors build one or more hyperplanes that divide the space in order to optimally partition data points into different classes [7]. Support vector machines are powerful tools for multiclass linear classification tasks, e.g., image segmentation, and have been employed in spine science for grading disc degeneration [8]. Decision trees link the values of the features from the input data to the possible outputs. The tree is divided into branches for each condition, with the terminal nodes representing the outcome of the decision [9]. Random forests utilize multiple decision trees, which are built on random subsets of the input features and generate an average of their prediction. Random forests limit the inherent susceptibility of overfitting of the decision trees. Artificial neural networks mimic the functional organization of the human brain by creating interconnecting neurons [10]. Information is transmitted and weighted through the neurons, which are organized in layers, and is processed through linear or nonlinear activation functions. Extending on the concepts of artificial neural networks, convolutional neural networks are based on the architecture of the animal visual cortex and are particular. Information from different groups of neurons, which are sensitive to particular image features, e.g., edge orientation, direction, shape, is weighted and combined to produce the final output [9]. Deep Learning constitutes a subfield of machine learning which utilizes methods involving multiple layers of processing units and can be practically viewed as multilevel artificial neural networks.

Using machine learning algorithms and cutting-edge deep learning architectures, artificial intelligence has recently altered the landscape of cancer research and medical oncology. Given the rising importance of personalized medicine, a wide range of artificial intelligence techniques have been widely employed to develop predictive algorithms, which model progression and response to treatment, ultimately informing effective and accurate decision making and improving outcomes of cancerous conditions. Continuous technical improvements have enabled computers to outperform expert human operators in tasks such as image classification, object detection, and landmark localization [11].

### 1.2. Neoplasms of the Spinal Canal

**Figure 1:**
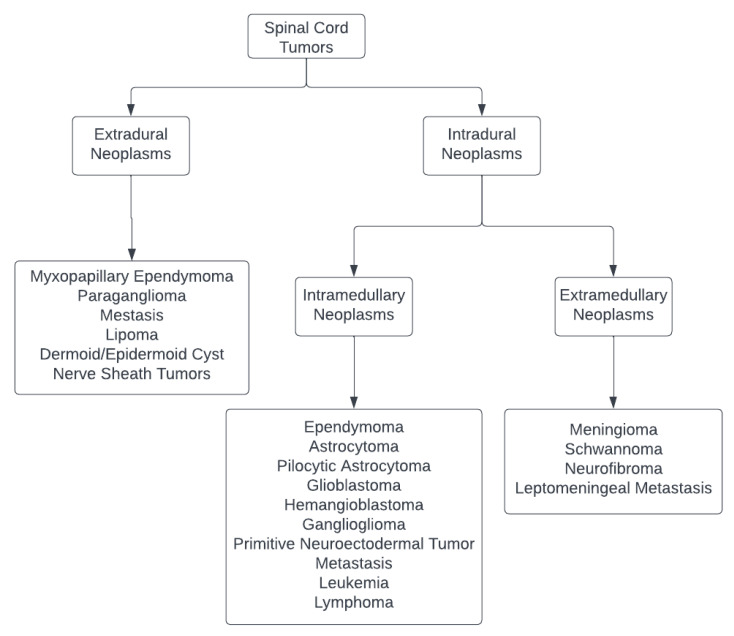
Classification of Spinal Cord Tumors [12,13].

The spinal canal may encompass a variety of tumors that develop within or affect the spinal cord, theca, and spinal nerves, which are frequently less aggressive and infiltrative than their cerebral counterparts. The spinal cord tumors per se can be studied as follows (Figure 1) [12,13]:

- Intramedullary lesions: Include spinal ependymoma, astrocytoma, pilocytic astrocytoma, glioblastoma, hemangioblastoma, ganglioglioma, primitive neuroectodermal tumors, metastasis, leukemia, or lymphoma;
- Intradural, extramedullary neoplasms: Include meningioma, nerve sheath tumors such as schwannoma or neurofibroma, and leptomeningeal metastasis;
- Tumors of the cauda equina and filum terminale: include myxopapillary ependymoma, paraganglioma, metastasis, lipoma, dermoid or epidermoid cysts, and nerve sheath tumors.

Before finalizing therapy options, precise knowledge of spinal cord tumor pathology is paramount. Tumor grading and categorization can also give patients and their families crucial prognostic information in addition to assisting with therapy choices. According to the 2021 World Health Organization central nervous system tumor grading/classification, tumors are graded within the types (rather than across different tumor types) with Arabic numerals (rather than Roman numerals employed in previous editions) [13]. According to the current classification, each lesion is given a grade between 1 and 4, with grade 1 being biologically benign and grade 4 being biologically the most malignant and having the worst prognosis. Generally, 88–90% of all primary spinal cord tumors develop in the extramedullary compartment, making them the most prevalent subtype [12].

## 2. Diagnosis

The importance of correctly diagnosing neoplastic lesions and classifying tumor patients into high or low risk groups has led many research teams from the biomedical and bioinformatics field to study the application of various machine learning methods, and this has followed suit in spinal cord tumor research.

### 2.1. Classification

Differentiating intramedullary spinal cord tumors from more common inflammatory demyelinating lesions poses a vital yet challenging task; despite their overlapping radiographic appearances, they have fundamentally different treatments and prognoses [14]. Zhuo et al. proposed a deep learning pipeline for classifying spinal cord lesions based on T2—weighted magnetic resonance images, which in some instances, even outperformed experienced neuroradiologists. They developed three two-classification models using two dimensional MultiResUNet and DenseNet121 networks, which compared tumor vs. demyelinating lesion, astrocytoma vs. ependymoma, and multiple sclerosis vs. neuromyelitis optica spectrum disorders. Their model achieved 96% accuracy in differentiating a tumor from an inflammatory lesion and 82% accuracy in classifying spinal astrocytoma versus ependymoma.

In order to better characterize the intramedullary spinal cord tumors based on the lesion’s position, size, and growth rate, segmentation of the lesion into tumor, edema, or cavity provides significant insight. To overcome manual segmentation’s time consuming and erroneous nature, Lemay et al. tailored an automated method, a cascaded architecture with U–Net-based models that segment tumors in a two-stage process involving locating and labeling [15]. The model initially identifies the spinal cord from multi contrast magnetic resonance images, generates bounding box coordinates as output, and crops the image to focus on an area of interest, thus mitigating class imbalance. The tumor is then divided into segments, and their model has shown a Dice score (a metric used to gauge machine learning model performance) of 61.8 ± 4.0% for the segmentation of tumors with a positive detection rate above 87%.

To mimic the routine clinical practice of incorporating patient clinical information to provide a radiological diagnosis, Liu et al. proposed a weighted fusion framework on magnetic resonance imaging to diagnose benign and malignant spine tumors at the patient level [16]. The weighted fusion framework included a combination of a tumor detection model, a sequence classification model, and a statistical module on age information. Their method facilitated the simultaneous delineation of tumor location, integration of the classification results of the tumor detection and the sequence classification models, aggregation of the results by majority voting, and finally, consideration of the patient’s age. Their weighted fusion framework model showed an accuracy of 82% as opposed to doctors’ 64–74% accuracy.

Artificial intelligence-based techniques, therefore, carry great potential to serve a confirmatory role for radiologists in the future, enabling focus on the altered image area and thus increasing workflow in neuroradiology [17]. Even though machine learning methods can improve our understanding of cancer progression, an appropriate level of validation is required to translate these methods into routine clinical practice.

Machine learning techniques, particularly deep learning approaches, have been employed to diagnose extramedullary tumors and differentiate among different types, such as schwannomas and meningiomas. Maki et al. used a deep learning framework to construct a convolutional neural network architecture on magnetic resonance images of patients [18]. The training images were processed by an orthopedic surgeon, who identified the minimal region containing the tumor and the anteroposterior border of the spinal canal. Radiologists reviewed the processed images, which were identical to the training dataset, in order to create equal competition between the convolutional neural network and the physicians. The model exhibited high diagnostic accuracy rates of over 80%, comparable to that of experienced radiologists. Ito et al. also employed a deep learning-based object detection system from imaging to develop an automated system for detecting spinal schwannomas [19]. T1- and T2-weighted images were manually labeled to facilitate training. The model was cross-validated by applying random transformations to the MRI images (e.g., flipping, scaling, etc.). This model yielded a high accuracy of over 93% using the T1- and T2-weighted images of the training dataset, comparable to identification by doctors. Cao et al. constructed a novel convolutional neural network-based deep learning model, incorporating multisource imaging data and prior clinical features [20]. Their model processed 11–12 slices extracted from contrast-enhanced T1 and T2 sequences, producing an attention map, which was then compared with the manually segmented lesion area. In classifying malignant nerve sheath tumors from spinal schwannomas, their approach outperformed conventional radiomic methods and radiologists’ assessment with a much greater accuracy (AUC of 0.95).

### 2.2. Molecular and Genetic Profiling

Apart from being utilized in diagnostic neuroradiology, machine learning techniques have also been used to understand the molecular genomics of spinal cord tumors. Jung et al. trained a random forest model with clinical and radiological data of 41 spinal cord glioma patients to predict the presence of H3 K27M mutations, which confer central nervous system tumors with poor prognosis [21]. Their random forest classifier showed that this histone gene mutation could be predicted with moderate discrimination (63.4% accuracy) based on clinical and radiological features. Pandey et al. utilized specific peptide motifs, conservation scoring schemes, Position Specific Scoring Matrices, and mutation matrices to develop a machine learning method (GlioBlastoma Multiforme Drivers) distinguishing between driver and passenger mutations in glioblastoma based on recurrence in patients with an accuracy of 73.6% [22]. Such methods can be applied to prioritize driver mutations and facilitate the identification of therapeutic targets.

Machine learning has also shown promise in the automated interpretation of histopathology of central nervous system tumors, identification of IDH1 mutation, MGMT promoter methylation, and 1p19q codeletion [23]. While most of these efforts have been focused on permanent sections of brain tumor specimens due to the larger amount of labeled data available and fewer artifacts, the results could very well be extrapolated to spinal cord tumors of similar histological features. Of late, machine learning approaches have also been applied for the automated diagnosis of intraoperative histopathologic specimens of central nervous system tumors [24]. A conventional machine learning approach, called multilayer perceptron, has achieved over 90% accuracy in classifying low grade glioma, high grade glioma, or nonglial tumors from digitally stimulated Raman Histology images (a label-free microscopy method based on Raman scattering to generate virtual slides of same diagnostic histopathologic features as permanent sections) [25]. Interestingly, this model could classify tumors using fresh, unstained intraoperative specimens, which were then converted into virtual hematoxylin- and eosin-stained slides, unveiling crucial diagnostic features. This demonstrates the prospect of deep neural network-based automated interpretation in assisting neuropathologists to boost diagnostic accuracy in complex or atypical cases.

Altogether, machine learning provides auxiliary information and may be used to deliver accurate diagnostic predictions and warning alerts by combining numerous data sets. Artificial intelligence is projected to progressively revolutionize clinical practice as its applications in radiological imaging, histopathology, and genomic information expand. Machine learning has demonstrated high diagnostic accuracy in spinal cord tumor prediction and distinction and continues to offer highly reproducible quantitative parameters that can even outperform qualitative assessments by experienced doctors.

## 3. Management

While the primary goal of surgery for spinal cord tumors is to restore neurological function and improve functional status and quality of life, the risks associated with surgery are balanced with achieving gross total resection to decrease the chance of tumor recurrence [26]. Khan et al. [27]. used machine learning to predict health-related quality of life outcomes after surgery for patients with mild degenerative cervical myelopathy. Their model employed seven different machine learning algorithms (random forests, classification trees, support vector machines, generalized additive models, generalized boosted models, partial least squares, and multivariable adaptive regression splines), which considered demographic and clinical factors and factors related to the surgical approach as predictor variables for the development of the algorithms [28]. There is scope for the development of similar algorithms for spinal cord tumors. Using the surgical approach as a predictor, surgeons can modify the approach beforehand, using the algorithm to find the optimal strategy for a given patient.

### 3.1. Surgical Planning

Artificial intelligence has the potential to individualize surgical planning and management for each patient. The heavily refined image processing aspect of machine learning can account for each patient’s anatomical variations, allowing for accurate reconstruction of relevant surgical anatomy. The use of machine learning for surgical planning is still in its infancy, and localization is key to a successful outcome. Localization, also known as object detection or classification, is an application of artificial intelligence that allows for identifying and labeling an object in an image [29]. Recent advances in localization have focused on the performance improvement of localization algorithms on suboptimal data. Jakubicek et al. [30]. developed a convolutional neural network trained on three-dimensional computerized tomography images, which consisted of three consecutive phases, including spinal axis determination, detection, and localization of intervertebral discs, and finally, identification and labeling of vertebrae. Their model correctly identified 87.1% of vertebrae using a data set that included distorted spines and incomplete spine scans, with a mean error of intervertebral disc localization of 4.4 mm. Algorithm training using incomplete data may increase the clinical utility and generalizability of localization in the future since clinical data are often not curated for processing by artificial intelligence. Nam KH et al. [31]. designed machine learning regression algorithms, using Google TensorFlow as the machine learning library, to predict the T-score of vertebrae, based on age, sex, and Hounsfield units. This approach reached a 92.5% accuracy in the test data set of 40 vertebrae, demonstrating the role of artificial intelligence in predicting osteoporotic vertebrae and ultimately informing surgical planning in spine surgery.

### 3.2. Resection

Benign lesions are more likely to exhibit an easy plane of dissection that facilitates resection; however, for infiltrative tumors, this may be difficult to establish. Marcus et al. [32] developed an artificial neural network that improved the prediction of surgical resectability in patients with glioblastoma multiforme. Artificial intelligence-based deep learning algorithms have been developed to facilitate surgeons to simultaneously maximize tumor removal while minimizing normal brain tissue displacement intraoperatively for glioblastoma multiforme [33]. The generated classification map is based on a four-step framework of blood vessel identification, parenchymal tissue detection, image classification, and morphological processing. Such multi-step concepts can be applied to infiltrative intramedullary spinal cord tumors such as astrocytomas, where it is difficult to distinguish between the normal spinal cord and tumor, facilitating maximal excision while simultaneously mitigating iatrogenic injury.

### 3.3. Intraoperative Diagnosis

A study by Khalsa SS et al. [24] applied Raman scattering to produce virtual hematoxylin and eosin slides without processing the tissue in real time. They used a convolutional neural network to identify 13 different brain tumors using intraoperative Simulated Raman Histology specimens with a diagnostic accuracy of 94.6%. Intraoperative specimens are burdened by artifacts during tissue preparation, variability in preparation techniques, and lack of digitalized data suitable for research. Developing a reliable automated model, which expedites surgical decision making based on intraoperative information, can improve the speed and accuracy of intraoperative preliminary diagnoses, guide treatment options, and inform prognosis at the time of surgery.

### 3.4. Navigation and Robotics

In modern spine surgery, neuronavigation and robotics are promising for machine learning applications. Current robotic surgical tools depend on physicians to pinpoint the area under interest. To decrease the time surgeons spend preparing for surgeries, healthcare institutions may turn to artificial intelligence in the form of predictive algorithms that will use real world data to put together preoperative plans. Three dimensional convolutional neural networks have shown promising results in aiding stereotactic radiation therapy planning [34]. Machine learning and deep learning methods have been used for automatic segmentation and contouring of tumors in head and neck oncology, saving clinicians time and producing good quality radiation planning contours [35]. Following the manual delineation of features of interest (gross tumor volume, clinical target volume, anatomical structures), the proposed algorithm was trained to learn physician contouring patterns and was able to predict high-risk clinical target volumes. The predicted contours could be applied clinically with minimal changes necessary. Implementing such models allows the tailoring of treatment plans according to individual patient and tumor characteristics, improving treatment accuracy and target delineation while reducing the time needed to select treatment plan parameters.

Computer-assisted navigation is widely utilized in operating rooms in the United States for operations ranging from spinal fusions to spinal tumor resection to complex spinal deformity. Current technology allows surgeons to construct a three-dimensional rendering of the spine, offering real time positional feedback and visualization of deeper structures, potentially avoiding iatrogenic injury. Intraoperative surgical robotics has the potential to implement artificial intelligence features and increase surgical precision and efficiency while avoiding complications as a result of human error and fatigue [36]. However, perioperative artificial intelligence platforms are yet to be published in the literature. It is important to note that although these devices could increase efficiency, speed, and accuracy in the operative room, they are ultimately tools that help the primary decision maker—the surgeon.

## 4. Postoperative Outcomes

Artificial intelligence models have been increasingly employed to predict outcomes and prognosis of spine disorders. Predicting clinical outcomes equips physicians with valuable information which can aid decision making, promote personalized medicine, and facilitate optimal patient management. Early attempts have demonstrated the applicability of machine learning in spine surgery [37,38,39], but with an evolving understanding of machine learning algorithms, progressively more advanced models have emerged. Although artificial intelligence techniques have been explored in other areas of spine surgery, such as spinal deformity surgery [40,41], precise predictive models for spinal tumors are somewhat scarce.

### 4.1. Predictive Models in Spinal Tumors

Given the rarity of spinal tumors, there is a paucity of literature unifying diverse predictors into an effective integrated risk model. Currently, the only published predictive machine learning model in spinal tumors was developed by Jin et al., who recruited a diverse set of clinical characteristics to predict postresection outcomes for intradural tumours. The proposed algorithm used a least absolute shrinkage and selection operator (LASSO) approach to classify discharge destination and 90-day readmission as binary outcomes based on input features, which included patient-specific and tumor-specific factors. Their integrated model outperformed other models, which employed only tumor-specific or patient-specific features, as it demonstrated superior discrimination and accuracy for non-home discharge and 90-day readmissions for patients undergoing surgery for intradural tumors [42]. Other non-machine learning calculators which provide risk stratification information for physicians have emerged. For example, neurological presentation, patient demographics, and incision length have been shown to be important predictors of nonroutine discharge, length of stay, readmission, and reoperation in patients undergoing surgery for intramedullary spinal cord tumors [43]. Similarly, Wang et al. developed a nomogram model and risk classification system for primary intramedullary spinal cord grade II/III ependymomas that accurately estimated individual overall survival probability [44]. However, although such non-artificial intelligence systems provide a potential framework for the development of machine learning algorithms, robust models are yet to be established. Nevertheless, the accurate prediction of postresection outcomes for spinal cord tumors, unifying heterogeneous clinical data, enables the transition to an era of personalized care.

### 4.2. Predictive Models in Spine Surgery

Invaluable information can be obtained by utilizing predictive models that have been developed to predict outcomes and complications in spine surgery, which are not specific to spinal tumors. Lee et al. created and validated a surgical site infection calculator, which provides a percentage prediction based on certain risk factors [45]. Their algorithm demonstrated fair predictive power, with a receiver operator character curve of 0.72, and is available online [46]. Similarly, other models have been devised which predict the likelihood of needing single versus multiple operative debridements once a surgical site infection has occurred [47]. Establishing such associations provides a foundation for machine learning models to develop upon. An artificial intelligence preoperative algorithm has been designed to predict the risk and automatically detect intraoperative vascular injury during lumbar surgery [48]. This model employed random forests to facilitate variable selection for the final algorithm training, which led to the identification of male sex, L4-L5 exposure, age, body mass index, surgery for infection, and diabetes as predictive features for intraoperative vascular injury. Following feature selection and development of five different supervised machine learning algorithms (random forest, support vector machine, stochastic gradient boosting, neural network, elastic-net penalized logistic regression), elastic-net penalized logistic regression achieved the best performance, with negative predictive values of 0.99 and 0.97 for automatic detection and preoperative prediction of vascular injury, respectively. Evidently, such tools provide significant insights that can inform decision making and shape patient management. Despite the promising potential exhibited by such models, further exploration of their application in spine surgery is warranted to validate their role in clinical practice.

### 4.3. Potential Imaging Biomarkers

Magnetic resonance imaging embodies an integral element of spinal disorders and offers an abundance of potential biomarkers that could complement clinical data to improve prognostic accuracy. In principle, identifying molecular and genetic profiles, such as platelet-derived growth factor in spinal ependymomas, can reveal treatment targets, expand treatment options, and ultimately improve patient outcomes [49]. Although extensive research has correlated genetic variants and histologic grades with survival in intracranial tumors, this information has not been universally translated to their spinal counterparts [50,51]. Contradictory evidence in the literature suggests that the predictive value of molecular profiles may be influenced by tumor location, hindering the development of machine learning models based on molecular data [50]. For example, KIAAA1549–BRAF mutations were seen in a higher frequency than BRAFV600E and other genetic aberrations in pediatric spinal low grade gliomas and may have been associated with lower death rates, but this difference only trended toward statistical significance [52]. Overall, further studies are required to investigate prognostic biomarkers and targetable genetic drivers in order to translate them into clinical practice (Table 1). [14,15,16,18,19,20,21,22,25,30,31,48].

## 5. Limitations

As techniques such as convolutional neural networks are based on a “black box” design, interpretability should be kept in mind. Although the automated nature of neural networks allows for the detection of patterns missed by humans, human scientists are left with little ability to assess how or why such patterns were discerned by the computer. It is important to note that artificial intelligence algorithms are only as good as the data that are used to train them. Most research methods utilize small data sets, so systemic and selection biases within the data set can impact the correlations and predictions generated by models. One must also consider the ethical and legal implications of artificial intelligence in surgery, such as patient privacy, confidentiality, and protection from cybercrime. Medicolegal regulation needs to be kept up to date and explicitly defined, as it may be challenging to hold an algorithm or its developers accountable in case of an adverse event. Prior to clinical implementation, machine learning models must be rigorously analyzed retrospectively and externally validated to ensure generalizability. Small scale prospective implementation, such as phases 1 and 2 of clinical trials, can help surgeons understand how these algorithms affect decision making among individuals and across different populations.

## 6. Conclusions

The rapidly expanding field of artificial intelligence, driven by major technological advances, embodies a promising tool in spine oncology, albeit still in its infancy. Future predictive models incorporating radiographic, pathologic, molecular, and oncologic data will provide more meaningful, precise, and unbiased assessments of survival and effectiveness of interventions. Importantly, extensive validation processes and quality assessments of the proposed algorithms are necessary to facilitate safe and effective translation to clinical practice. Applying artificial intelligence to complement human decision making, and not replace it, should be encouraged to unlock its full potential and enable the transition to an era of data-driven personalized medicine in spinal cord tumors.

## Figures and Tables

**Table 1:** Summary of publications studying machine learning application in spinal cord tumors or spinal surgery.

Name	Machine Learning Algorithm	Clinical Context	Application	Performance	Sample Size (Patients)
Zhuo et al. 2022 [14]	Deep Learning— MultiResUNet and DenseNet121	Astrocytoma, Ependymoma, Multiple Sclerosis, Neuromyelitis Optica Spectrum Disorders	Diagnosis, Classification	Tumor vs. Inflammatory Lesion—Accuracy 96%; Astrocytoma vs. Ependymoma—Accuracy 82%	490
Lemay et al. 2021 [15]	Deep Learning—Convolutional Neural Network	Astrocytoma, Ependymoma, Hemangioblastoma	Diagnosis, Classification, Segmentation	Positive Detection Rate 87%	343
Liu et al. 2022 [16]	Deep Learning—Weight Fusion Framework	Benign vs. Malignant	Diagnosis, Classification	Benign vs. Malignant—Accuracy 82%	585
Maki et al. 2020 [18]	Deep Learning—Convolutional Neural Network	Schwannoma, Meningioma	Diagnosis, Classification	Schwannoma vs. Meningioma—Accuracy 80%	84
Ito et al. 2021 [19]	Deep Learning—Google Tensorflow	Schwannoma	Diagnosis, Detection	Accuracy 93%	50
Jung et al. 2019 [21]	Random Forest	Glioma	Diagnosis, Classification	H3K27M Detection—Accuracy 63.4%	41
Pandey et al. 2022 [22]	-	Glioblastoma	Diagnosis, Classification	Driver vs. Passenger Mutation—Accuracy 74%	-
Orringer et al. 2017 [25]	Multilayer Perceptron	Glioma, Metastasis, Meningioma, Lymphoma, Medulloblastoma	Diagnosis, Classification	Overall Accuracy 90%	101
Jakubicek et al. 2020 [30]	Deep Learning—Convolutional Neural Network	Vertebral Identification	Surgical Planning, Classification	Vertebral Identification Accuracy 87%	421
Nam KH et al. 2019 [31]	Deep Learning—Google Tensorflow	Osteoporotic Vertebrae	Surgical Planning, Classification	Osteoporotic Vertebrae—Accuracy 92%	70
Karhade et al. 2021 [48]	Natural Language Processing	Intraoperative Vascular Injury	Surgical Planning	Preoperative Prediction of Vacular Injury—Negative Predictive Value 0.99	1035

## Data Availability

Data sharing not applicable. No new data were created or analyzed in this study. Data sharing is not applicable to this article.
